# Swept-Source Optical Coherence Tomography Angiography According to the Type of Choroidal Neovascularization

**DOI:** 10.3390/jcm8091272

**Published:** 2019-08-22

**Authors:** Joon Hyung Yeo, Hum Chung, Jee Taek Kim

**Affiliations:** Department of Ophthalmology, College of Medicine, Chung-Ang University, Seoul 06973, South Korea

**Keywords:** optical coherence tomography angiography, neovascular age-related macular degeneration, choroidal neovascularization

## Abstract

We analyzed and compared the sensitivity of choroidal neovascularization (CNV) detection according to CNV type in patients with active neovascular age-related macular degeneration (AMD) using swept-source optical coherence tomography (OCT) angiography (OCTA). A retrospective chart review was performed in patients with neovascular AMD. OCTA images were classified into three groups: Group A (well-circumscribed vascular complex); Group B (moderately circumscribed vascular complex); and Group C (poorly circumscribed vascular complex), according to CNV appearance. Demographic characteristics, OCT parameters, neovascularization subtypes, and OCTA image quality were analyzed to determine the effect on visualization of the neovascular complex. A total of 130 patients with CNV secondary to active neovascular AMD were analyzed. Among them, 52 eyes from 47 patients were included in the study. Eighteen eyes (34.6%) were classified into Group A, 24 (46.2%) into Group B, and 10 (19.2%) into Group C. Statistical analysis showed no significant differences in demographic characteristics or OCT parameters between the three groups. Overall sensitivity of active CNV detection was 80.7% (42/52 eyes). In 73.5% (25/34) of eyes with type 1 CNV (sub-retinal pigment epithelial type), 100.0% (9/9) of eyes with type 2 CNV (sub-retinal type), and 88.9% (8/9) of eyes with type 3 CNV (retinal angiomatous proliferation type), the vascular complex was well visualized on OCTA. OCTA provides adequate noninvasive imaging of CNV in patients with neovascular AMD, which may assist in CNV diagnosis and activity monitoring. In particular, type 2 CNV was well detected in OCTA in comparison with type 1 and type 3 CNV.

## 1. Introduction

Neovascular age-related macular degeneration (AMD), characterized by choroidal neovascularization (CNV), is a leading cause of vision impairment in developed countries. Severe visual loss of AMD may occur due to CNV and its consequences: exudation, bleeding, and disciform scar [1]. 

Fluorescein angiography (FA) has been used as the gold standard to visualize CNV in the retina [2,3]. Indocyanine green angiography (ICGA) has been also used to detect polypoidal lesions in the choroid [4]. These angiography examinations show considerable sensitivity and specificity for the detection of CNV or polyp. Moreover, a recently developed confocal type of angiography (Heidelberg Retina Angiograph 2, Heidelberg Engineering, Heidelberg, Germany) showed detailed vascular abnormalities in the macula. However, FA and ICGA, which both use intravenous dye injection, are inevitably associated with several adverse effects, ranging from mild nausea and rash to serious anaphylactic shock.

CNV has been classified into a predominantly classic type, a minimally classic type, and occult type according to the FA findings. Recently, the evolution of optical coherence tomography (OCT) has provided a new classification for CNV according to the localization of the new vessel complex: type 1 of the sub-retinal pigment epithelial (RPE) type; type 2 of the sub-retinal type; and type 3 of the retinal angiomatous proliferation type [5].

OCT angiography (OCTA) is a recently developed imaging technique that employs an amplitude or phase decorrelation algorithm with high-frequency and dense volumetric scanning, allowing for noninvasive direct visualization of the chorioretinal vasculature in vivo [6,7,8,9,10]. The noninvasive characteristics of OCTA facilitate repeated evaluation. However, the CNV complex is sometimes not detectable using OCTA in clinical practice, even in eyes with active neovascular AMD.

The purpose of this study was to assess and compare the sensitivity of swept-source OCTA (SS-OCTA) to visualize CNV lesions according to CNV type and analyze the factors that affect visualization of CNV in patients with active neovascular AMD. 

## 2. Materials and Methods

### 2.1. Patients

This study was approved by the Institutional Review Board of Chung-Ang University Hospital in Seoul, Korea and followed the tenets of the Declaration of Helsinki. In this retrospective observational case series study, patients who underwent OCTA using the SS-OCTA system of the commercially available DRI Triton OCT device (Topcon, Tokyo, Japan) between November 2015 to July 2016 at the Retina Service of the Ophthalmology Department of Chung-Ang University Hospital, were evaluated. 

A retrospective chart review was performed in patients with neovascular AMD and clinical data were collected including patient demographics, best-corrected visual acuity (logMAR), intraocular pressure, refractive errors, and history of intravitreal anti-vascular endothelial growth factor (VEGF) treatment. Inclusion criteria were active subfoveal CNV lesions secondary to neovascular AMD as determined by OCT, FA, and ICGA, regardless of previous treatment. The following exclusion criteria were applied: subretinal hemorrhage or fibrotic scar; geographic atrophy; previous retinal surgery; previous history of focal laser or photodynamic treatment; and/or the presence of other retinal diseases including diabetic retinopathy, retinal vein occlusion, and epiretinal membrane. Eyes with a mixed form of types 1 and 2 CNV, juxtafoveal CNV that extended beyond the 3 mm × 3 mm scan area, low quality OCTA images (image quality index below 50), and/or with severe media opacity and poor fixation due to low visual acuity were also excluded. 

### 2.2. Scanning Protocol of Optical Coherence Tomography (OCT) and OCT Angiography (OCTA)

OCT and OCTA images were acquired with the DRI Triton OCT device with a full-spectrum amplitude decorrelation angiography algorithm (version 1.16; Topcon, Tokyo, Japan), with a wavelength of 1050 nm and a scan speed of 100,000 A-scans per second, which yielded an axial resolution of 8 μm and a depth of 2.4 dB/mm. OCT B-scan imaging was performed with a 6 × 6 mm cube scan and a 9 mm five-line cross-scan. OCTA imaging was performed at the 3 × 3 mm center of the fovea with a resolution of 320 × 320 pixels. The OCTA algorithm was based on detecting the degree of motion between consecutive OCT images. Briefly, OCT B-scan images were collected at the same transverse location four times. These four images were then registered with each other using a registration algorithm. The degree of motion was then calculated to allow for the extraction of blood flow. Automated layer segmentation boundaries were manually adjusted to best visualize the neovascular complex on the en face projection angiography images. 

### 2.3. Classification of Choroidal Neovasculization (CNV)

**Figure 1:**
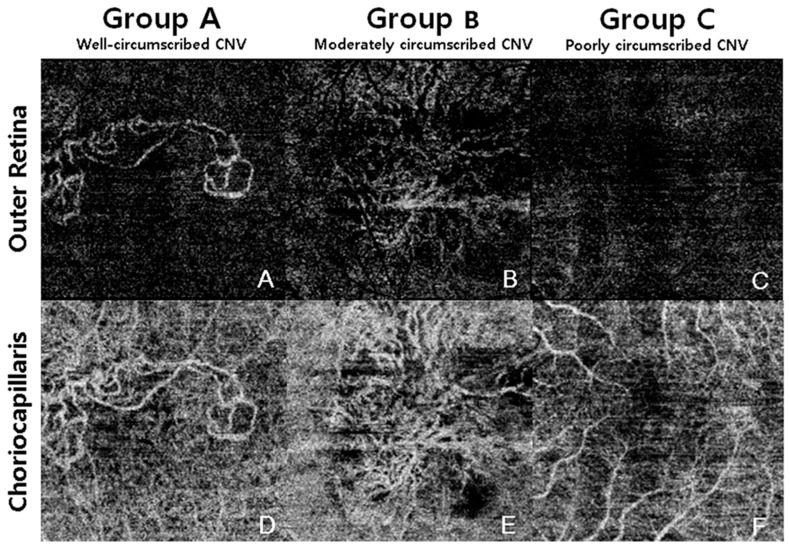
Classification of choroidal neovascularization (CNV) group according to the visualization of CNV in optical coherence tomography angiography. Group A was defined as CNV, of which over 80% of the contour could be demarcated (well-circumscribed vascular complex); Group B, where 50%–80% of the contour could be demarcated (moderately circumscribed vascular complex); and Group C, where less than 50% of the contour could be demarcated (poorly circumscribed vascular complex).

CNV lesions were classified into three groups according to anatomic localization as determined with multimodal imaging employing FA, ICGA, and OCT. The three groups were: type 1 neovascularization with or without polypoidal lesion (CNV located under the RPE); type 2 neovascularization (CNV located above the RPE); and type 3 neovascularization (intra-retinal) [5]. Polypoidal choroidal vasculopathy (PCV) was classified as a subtype of type 1 neovascularization [11]. OCTA images were classified into three groups according to CNV appearance after masking the type of CNV: Group A was defined as CNV, of which over 80% of the contour could be demarcated (well-circumscribed vascular complex); Group B, where 50%–80% of the contour could be demarcated (moderately circumscribed vascular complex); and Group C, where less than 50% of the contour could be demarcated (poorly circumscribed vascular complex) (Figure 1). A detection score system was subsequently determined, composed of three points for eyes in Group A, two points for eyes in Group B, and one point for eyes in Group C. The type of CNV and selection of proper visualization subgroups were assessed by two blinded readers (J.H.Y. and J.T.K.), and a third senior reader (H.C.), who resolved cases of disagreement between the first two readers. The activity of CNV was also determined by the recurrence of intraretinal, subretinal, and sub-RPE fluid. OCT parameters including the height or width of the pigment epithelium detachment (PED), subfoveal choroidal thickness, center fovea thickness, height of the subretinal fluid, and CNV size were measured by two readers (J.H.Y and J.T.K.) using the built-in caliper tool of the OCT device (DRI Triton; Topcon, Tokyo, Japan).

### 2.4. Statistics

Statistical analyses were performed with SPSS, version 23.0 (SPSS Inc., Chicago, IL, USA), using Fisher’s exact test, the Chi square test, one-way ANOVA, and/or the Kruskal–Wallis analysis with post-hoc analysis. Statistical significance was defined by *p* < 0.05.

## 3. Results

A total of 130 patients (172 eyes) with CNV secondary to active neovascular AMD visited our clinic. Among them, 52 eyes from 47 patients meeting our inclusion and exclusion criteria were included in the study. The demographic and clinical characteristics of these patients are summarized in Table 1. The mean age was 73.3 ± 8.9 years (range: 52–89 years), and 24 of them (51.1%) were female. The best corrected visual acuity was 0.52 ± 0.38 (range: 0–1.9 logMAR). Seven out of 52 eyes (13%) were treatment-naive cases, while 45 eyes (87%) had undergone previous treatment with intravitreal anti-VEGF agents. In previously treated eyes, the mean number of intravitreal anti-VEGF injections was 6.6 ± 4.5 and the mean duration after initial diagnosis was 16.9 ± 14.7 months. On the OCT B-scan, the mean maximal height of PED was 167.0 ± 145.5 µm. The mean maximal width of PED was 2353.0 ± 1374.2 µm. A total of 24 eyes showed subretinal fluid, with a mean subretinal fluid height of 51.6 ± 71.3 μm.

In OCTA imaging, 18 out of 52 eyes were classified as Group A (well-circumscribed, 34.6%); 24 of 52 eyes were classified as Group B (moderately circumscribed; 46.2%); and 10 of 52 eyes were classified as Group C (poorly circumscribed; 19.2%). Patient demographics and OCT parameters including height or width of PED, CNV area, choroidal thickness, and central fovea thickness did not show significant differences between groups (Table 2).

Using multimodal imaging, eyes with neovascular AMD were divided into three subtypes of CNV. Thirty-four eyes were classified as type 1 CNV, nine as type 2 CNV, and nine as type 3 CNV. OCT parameters, according to the CNV subtypes, are shown in Table 3. Eyes with type 1 CNV had significantly thicker subfoveal choroidal thickness compared to type 3 CNV (232.5 ± 101.1 μm vs. 139.6 ± 51.1 μm, *p* = 0.019; Kruskal–Wallis test with post-hoc analysis). The CNV area in the OCTA was significantly smaller in eyes with type 3 CNV than in eyes with type 2 CNV (0.51 ± 0.83 mm^2^ vs. 3.52 ± 3.41 mm^2^, *p* = 0.005; Kruskal–Wallis test with post-hoc analysis).

Eyes in Groups A, B, and C were subclassified according to CNV type (Table 4). Eyes in Group A and Group B were considered as being detected with CNV lesion. Thus, the overall sensitivity of CNV detection was 80.7% (42/52); that of type 1 CNV was 73.5% (25/34), type 2 CNV was 100% (9/9); and type 3 CNV was 88.9% (8/9). Detection score was higher for type 2 CNV (2.56) than for type 1 CNV (2.03) or type 3 CNV (2.22), although the difference was not statistically significant (a higher score suggests better visualization). 

In eyes with type 1 CNV, the OCT parameters were analyzed between the CNV groups (Table 5). However, no statistically significant differences between the groups were revealed. 

**Figure 2:**
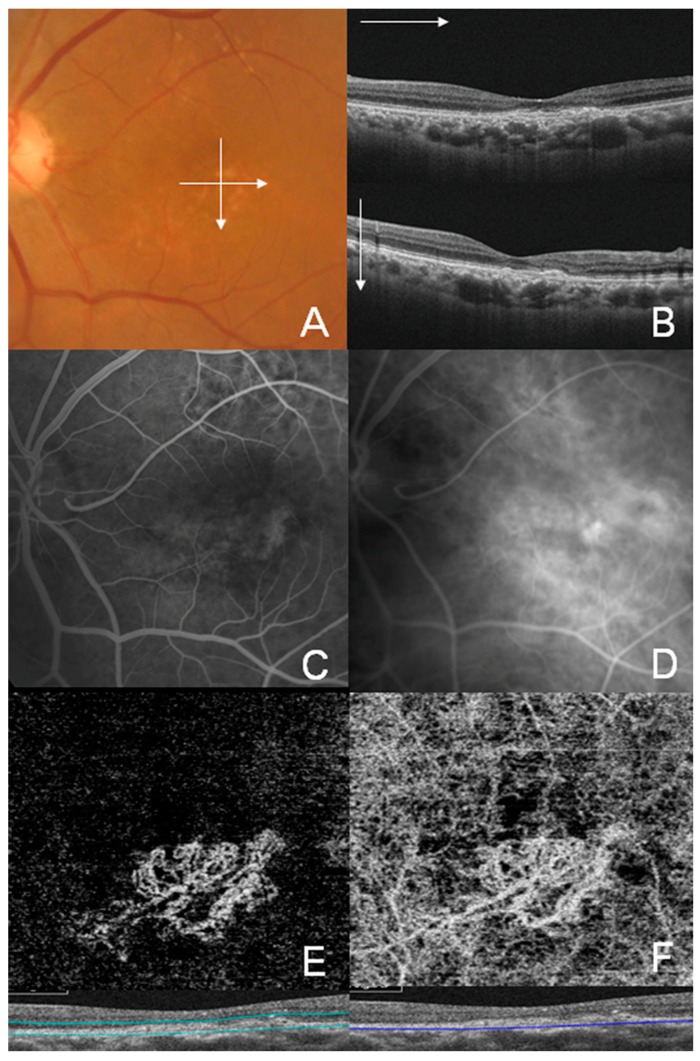
Multimodal imaging of Group A (well-circumscribed) type 1 choroidal neovascularization (CNV) in a 72-year–old male. (**A**) Color fundus photograph; (**B**) Swept-source optical coherence tomography (OCT) B-scan image of the fovea showing shallow vascularized pigment epithelial detachment with choroidal thickening. Double-layer sign with a fibrovascular notch suggesting polypoidal choroidal vasculopathy; (**C**) Early phase fluorescein angiography (FA) showed ill-defined CNV; (**D**) Late-phase FA showed ill-defined vascular leakage; (**E**) OCT angiography (OCTA) image of the outer retinal layer of a hyperflow seafan-like pattern lesion; and (**F**) OCTA images of the choriocapillaris.

**Figure 3:**
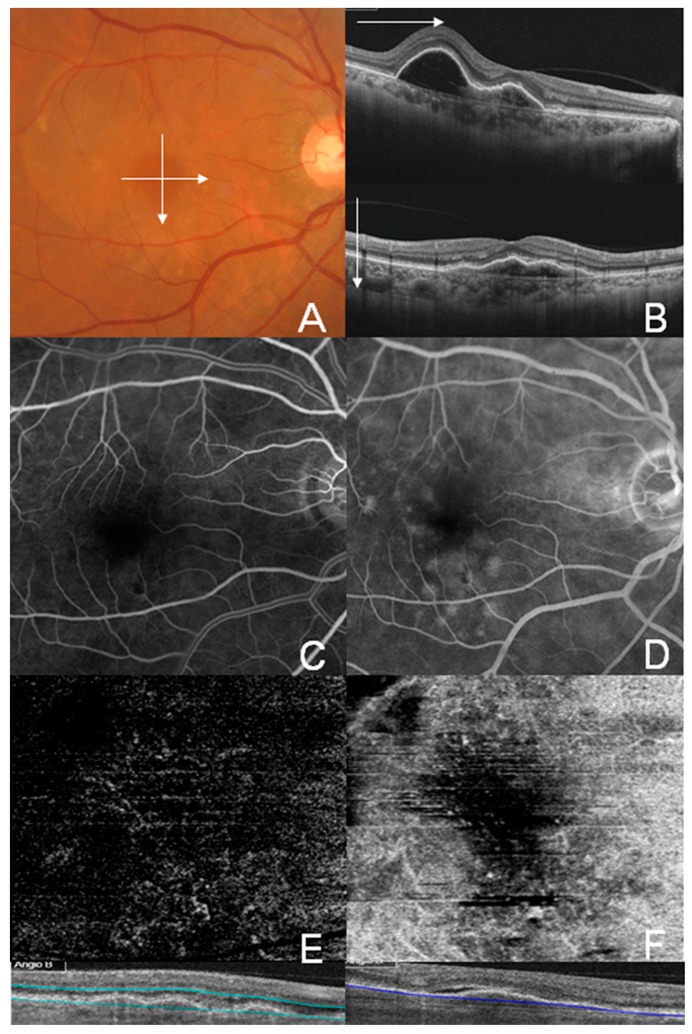
Multimodal imaging of a 63-year-old female showing type 1 choroidal neovascularization (CNV), classified as Group C (poorly circumscribed CNV). (**A**) Color fundus photograph showing a broad orange nodule on the posterior pole corresponding to a large pigment epithelial detachment (PED); (**B**) Swept-source optical coherence tomography (OCT) B-scan image on the horizontal and vertical 6 mm of the fovea showing vascularized and serous PED; (**C**,**D**) Early and late phase images of the fluorescein angiography showed ill-defined occult type CNV. (**E**,**F**) OCT angiography images of the outer retina layer (**E**) and choriocapillaris (**F**) showed poorly circumscribed vascular contour.

**Figure 4:**
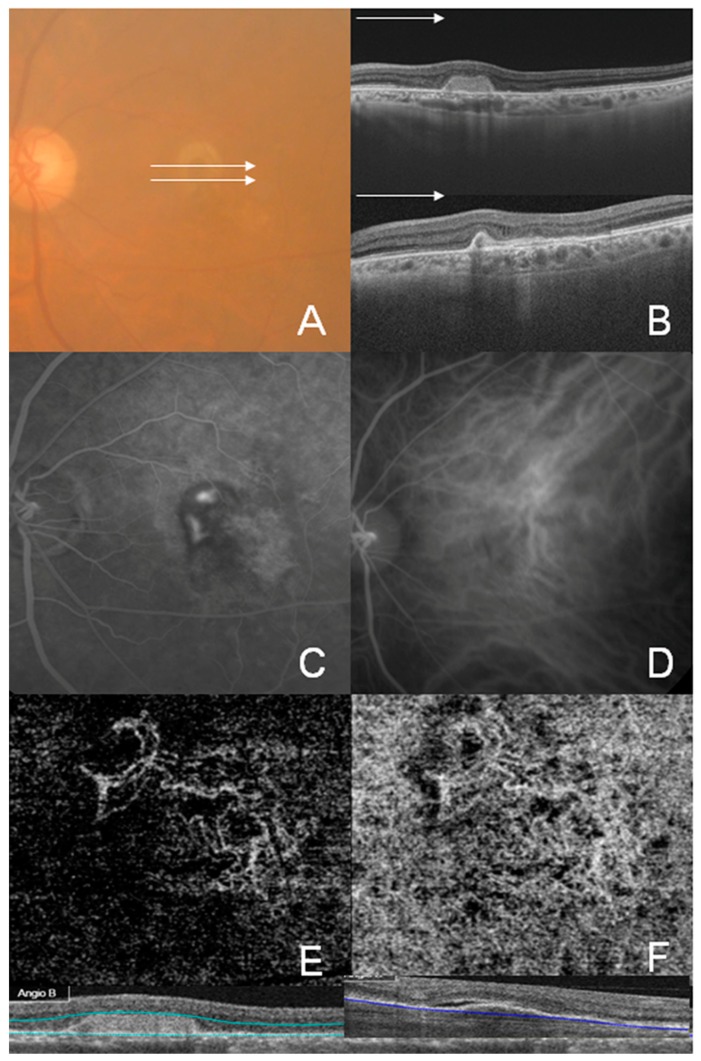
Multimodal imaging of a 60-year-old male showing well-circumscribed (Group A) type 2 choroidal neovascularization (CNV). (**A**) Color fundus photograph obscured due to grade 3 nuclear sclerosis-cataract; (**B**) Swept-source optical coherence tomography (OCT) B-scan image of the horizontal 6 mm of the central fovea (white lines on fundus photograph). (**C**,**D**) Early phase image of fluorescein angiography (**C**) and indocyanine green angiography (**D**) showing hyper-fluorescent classic CNV lesion. (**E**,**F**) OCT angiography images of the outer retina layer (**E**) and choriocapillaris (**F**) showing a well-circumscribed CNV network.

**Figure 5:**
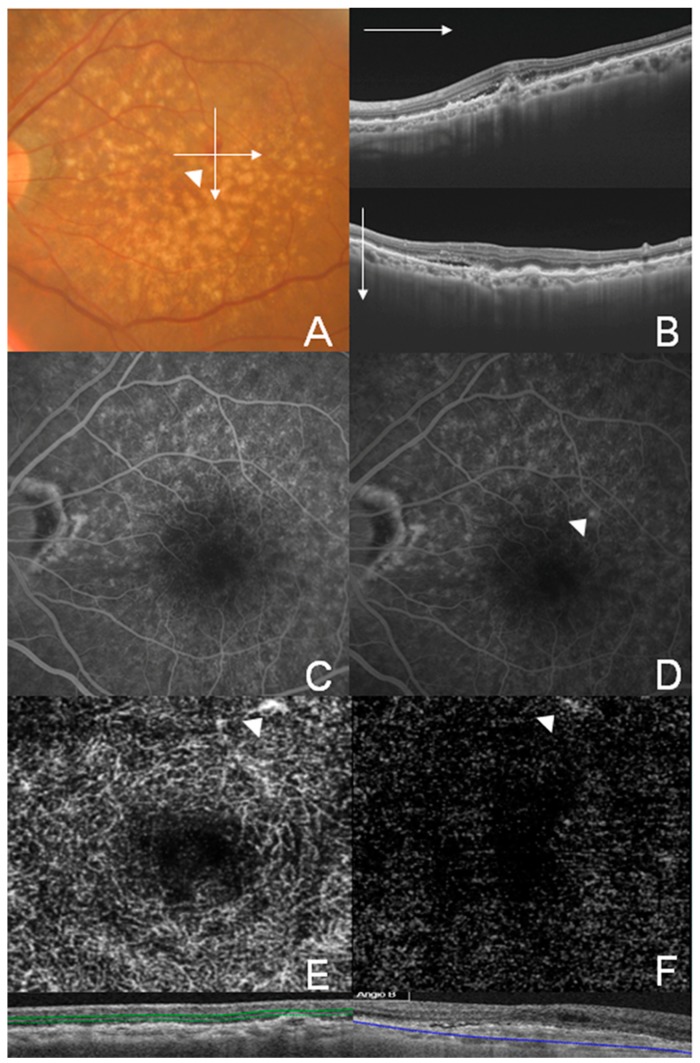
Multimodal imaging of the left eye of a 63-year-old female showing type 3 choroidal neovascularization (CNV). (**A**) Color fundus photograph showing multiple drusen and intraretinal hemorrhage associated with CNV; (**B**) Swept-source optical coherence tomography (OCT) image through the white line drawn on color photograph showing drusenoid retinal pigment epithelium detachment; (**C**,**D**) Early (**C**) and late (**D**) phase of the fluorescein angiography (FA). Type 3 CNV is not clearly identifiable with FA; (**E**,**F**) OCT angiography images showing intraretinal vascular tuft of the deep retinal capillary plexus layer (**E**) and outer retina layer (**F**). The white arrowhead indicates a small but distinct type 3 CNV lesion.

Representative cases of well-circumscribed (Group A) type 1 CNV, poorly circumscribed (Group C) type 1 CNV, well-circumscribed (Group A) type 2 CNV, and well-circumscribed (Group A) type 3 CNV are shown in Figure 2, Figure 3, Figure 4 and Figure 5, respectively. Specifically, Figure 2 shows the fundus photography, OCT, FA, and ICGA images of a 72-year-old man with type 1 CNV in the left eye. The ICGA image demonstrates the presence of a subfoveal polypoidal vascular lesion, showing a hot spot in the region of CNV. The OCTA image also illustrates a small, well-circumscribed subfoveal CNV in the outer retina slab and choriocapillaris slab. Figure 3 shows poorly circumscribed type 1 CNV in the eyes of a 63-year-old female. The FA images showed occult type characteristics. Figure 4 shows the fundus photography, OCT, FA, ICGA, and OCTA images of a 60-year-old male with type 2 CNV. The FA image demonstrates subfoveal CNV, showing hyperfluorescence early with later leakage in the region of CNV. The CNV lesion is evident in the outer retina slab and choriocapillaris slab in the OCTA images. Figure 5 demonstrates type 3 CNV in the left eye of a 63-year-old female, with multiple large confluent soft drusen observed in the fundus photography, OCT, FA, and OCTA images. A dilated vascular lesion was prominent in the outer retina slab, but not in the choriocapillaris slab of the OCTA images.

## 4. Discussion

This study evaluated the potential to visualize active CNV using commercially available SS-OCTA. The overall sensitivity for active CNV was 80.7% (42/52 eyes), and type 2 CNV showed a higher sensitivity regarding detection (100%) compared to type 1 CNV (73.5%) and type 3 CNV (88.9%).

OCTA is a new imaging method that provides a three-dimensional, depth-resolved image and can also detect the microvasculature of the retina and choroid. The first device and algorithms of OCTA were originally developed using spectral-domain OCT (SD-OCT). SD-OCT uses an interferometer with a low coherence light source and measures the interference spectrum with a spectrometer. However, the inherent technical issues result in decreased resolution through the choroidal tissue [12]. In contrast, SS-OCT provides potential advantages for imaging the choroid using longer wavelengths, which can penetrate deeper tissues, especially RPE, due to better signal penetration and less washout of interference fringes from flow velocity [7,13].

A previous study using SD-OCTA reported a sensitivity of 50% and a specificity of 91%, respectively, for CNV detection in 30 eyes with neovascular AMD [14]. In contrast, another study reported that SS-OCTA could visualize 16 of 17 eyes with active CNV, with 94% sensitivity for CNV detection [9]. Moreover, another recent publication has reported that SS-OCTA using a 1050 nm wavelength provided a more accurate representation of CNV lesion and significantly larger CNV area when compared to SD-OCTA using an 840 nm wavelength [15]. These studies suggest that SS-OCTA is more sensitive than SD-OCTA for CNV detection, although these previous publications were not prospective nor controlled studies.

In this study, we tried to analyze the factors that affect the visualization of CNV. First, CNV lesions were classified as types 1, 2, and 3 CNV using multimodal imaging. Next, OCTA images were classified into groups A, B, and C after segmentation adjustment. We tried to quantify the visualization of OCTA according to pre-set criteria. Table 4 shows the 3 × 3 cross-tabulation according to CNV type and CNV detection group. However, it is difficult to analyze statistical significance with a 3 × 3 table. Thus, we used a detection score system to quantify the detection score and analyze the statistical significance. Groups A, B, and C were given scores of 3, 2, and 1, respectively. The scores between the three CNV groups did not show statistically significant differences. However, when we compared the detection score between type 1 CNV and type 2 CNV after the exclusion of type 3 CNV, the score of type 2 CNV was higher than the score of type 1 CNV (independent-*t* test, *p* = 0.04). Furthermore, type 2 CNV showed a higher percentage in Group A (56%) than type 1 CNV (29%) (McNemar’s test, *p* = 0.001).

Farecki et al. have also reported that type 2 CNV demonstrated a sharper demarcation from the surrounding vasculature than type 1 CNV [16]. Using SD-OCTA, Lumbroso et al. detected CNV in all cases of five eyes with naive type 2 CNV and were able to successfully follow the lesion [17]. Ameen et al. detected CNV in all cases of 14 consecutive type 2 CNV patients and described the lesion as medusa-shaped or glomerulus-shaped lesion [18]. Parravano et al. also detected and followed the CNV lesion in 12 eyes of 12 patients with previously treated type 2 CNV [19]. In addition, this anatomic location of type 2 CNV (above the RPE) leads to a well-defined or easily recognizable “classic” lesion by FA. The good visibility noted when using FA for type 2 CNV corresponded with the findings of an evaluation using OCTA.

Type 1 CNV is neovascularization occurring beneath the RPE layer, which is the most common form of CNV in AMD [20]. These type 1 CNV cases are usually described as poorly defined or occult type CNV with FA. This inadequate visibility using FA in type 1 CNV is also in accordance with the findings of an examination using OCTA. Kuehlewein et al. reported that CNV could be identified in 75% of 25 patients with type 1 CNV [21], which was in agreement with our findings. Moreover, FA is insufficient in differentiating type 1 from type 2 CNV as FA generally fails to show whether CNV is above or below the RPE, or detect the polyp and branching vascular networks in polypoidal choroidal vasculopathy [22,23]. Thus, multimodal imaging using FA, ICGA, and OCT is required to distinguish between type 1 and type 2 CNV or to detect PCV.

Type 3 CNV is characterized by small intraretinal neovascularization [5]. This neovascular tuft is located most superficially in the retina. FA shows focal early hyperfluorescence and late leakage. Additionally, in OCTA, type 3 CNV can be visualized as a discrete high-flow linear structure extending from the middle retinal layers into the deep retina and occasionally past the RPE (Figure 5). In this study, type 3 CNV was visualized in 88.9% (8/9) of patients using SS-OCTA (Figure 5). However, only three eyes of type 3 CNV, especially in naive cases, were classified into Group A. As type 3 CNV showed a rapid response to anti-VEGF therapies, which was associated with the anatomic location of type 3 CNV, treated collapsed vascular tuft was not detected using OCTA. These findings are consistent with those from a previous study using SD-OCTA. Phasukkijwatana et al. reported that type 3 lesion was detected in all 17 naive eyes and was undetectable with OCTA in five of them after injection [24]. Kuehlewein et al. reported that type 3 CNV was identified in only 34% (10 of 29) of eyes [25]. In addition, visualization of type 3 CNV requires manual adjustment because of the variable location of the lesion from the superficial to deep retina. Therefore, type 3 CNV, especially with a history of anti-VEGF treatment, should be carefully examined for diagnosing and monitoring CNV. 

This study has several limitations. First, this study is a retrospective observational cross-sectional case series. Ideally, the scan should be performed in a prospective manner in neovascular AMD eyes with unknown CNV lesions. However, CNV is occasionally not detected using OCTA in clinical practice, even in eyes with active neovascular AMD. Therefore, we investigated the sensitivity of SS-OCTA to “visualize” CNV lesions according to CNV type, not to detect CNV *per se*. Second, this study included a relatively small sample size. In particular, only 18 eyes were included: nine for type 2 CNV and nine for type 3 CNV. Thus, prospective studies with a larger sample size are required to confirm the results of this study and elucidate the factors that affect the visualization of CNV. Third, almost all included patients had been previously treated with anti-VEGF. Therefore, it is important to further analyze the use of OCTA in naive AMD eyes. Fourth, the segmentation error was manually adjusted in this study. Segmentation errors commonly occur in edematous retina with exudation, hemorrhage, and PED, especially in naive eyes. Manual adjustment of the segmentation boundary may increase the sensitivity of CNV detection. Thus, the CNV lesion in the slab of the outer retinal layer does not suggest type 2 CNV above RPE, in this study. Moreover, the CNV lesion in the slab of choriocapillaris does not suggest type 1 CNV under RPE. However, manual processing is time-consuming and can only be rarely performed in clinical settings. Advanced algorithms for segmentation may increase the detection rate of CNV without manual processing in the future. Fifth, the specificity of CNV detection was not analyzed in this study. The detection of CNV does not necessarily accurately represent the activity of CNV in neovascular AMD, because detection does not indicate leakage from CNV. This suggests that the specificity of OCTA is usually not high. We analyzed the sensitivity and detection rate of CNV only in active AMD patients who received anti-VEGF treatment due to intraretinal cyst or edema, because we sometimes could not detect CNV even in eyes with active AMD. Finally, the association between OCTA visualization and OCT parameters was not analyzed. However, a larger area of CNV in type 2 CNV in this study might be associated with a higher sensitivity of CNV. Further research is required to analyze specificity as well as sensitivity in a larger number of patients.

In conclusion, this study showed the clinical utility of SS-OCTA during the follow up of CNV lesion in neovascular AMD. OCTA is a promising imaging technique for monitoring CNV, especially in patients with type 2 CNV. Updated algorithms and techniques of OCTA may enhance the detection rate in the future.

## Figures and Tables

**Table 1:** Patient demographics and parameters of optical coherence tomography (OCT) and OCT angiography in patients with neovascular age-related macular degeneration.

Parameters	Data
No. of patients (no. of eyes)	47 (52)
Sex (female, %)	24 (51.1)
No. of treatment-naive eyes (%)	7 (13)
No. of intravitreal injection	6.6 ± 4.5
Age, years	73.3 ± 8.9
Mean BCVA, logMAR	0.52 ± 0.38
Disease duration, months	16.9 ± 14.7
PED max height, μm	167.0 ± 145.5
PED max width, μm	2353.0 ± 1374.2
Subfoveal choroidal thickness, μm	216.7 ± 98.1
Central fovea thickness, μm	260.0 ± 142.7
CNV area, mm^2^	1.6 ± 2.0
SRF height, μm	51.6 ± 71.3
SRF existence, no. of eyes	24
Appearance of CNV (no. of eyes, %)	
Group A; well-circumscribed	18 (34.6)
Group B; moderately circumscribed	24 (46.2)
Group C; poorly circumscribed	10 (19.2)
Detection sensitivity, %	80.70
CNV detection score, points	2.15 ± 0.72

BCVA: best-corrected visual acuity; PED: pigment epithelium detachment (serous or vascularized); CNV: choroidal neovascularization; SRF: subretinal fluid. Data are expressed as mean ± SD.

**Table 2:** Classification of eyes with neovascular age-related macular degeneration according to detection group of choroidal neovascularization (CNV).

	Group A (Well Circumscribed)	Group B (Moderately Circumscribed)	Group C (Poorly Circumscribed)	*p*-Value
No. of eyes	18	24	10	-
Mean age, years	74.5 ± 2.0	72.8 ± 9.4	71.9 ± 9.3	0.687 *
Sex (female, %)	10 (55.6)	9 (37.5)	5 (50)	0.491 ^†^
BCVA, logMAR	0.63 ± 0.40	0.45 ± 0.30	0.51 ± 0.49	0.279 ^‡^
IOP, mmHg	14.2 ± 3.3	15.0 ± 3.1	15.8 ± 4.0	0.221 ^‡^
No. of treatment naive eyes	2	5	0	0.326 ^§^
No. of intravitreal injection	4.2 ± 3.4	6.2 ± 5.2	6.2 ± 4.7	0.838 ^‡^
Disease duration, months	14.5 ± 14.1	17.9 ± 15.6	18.9 ± 14.6	0.524 ^‡^
PED max height, μm	167.2 ± 167.3	163.6 ± 144.9	174.7 ± 115.5	0.515 ^‡^
PED max width, μm	2493.4 ± 1364.6	2425.7 ± 1425.2	1926.0 ± 1317.7	0.421 ^‡^
Choroidal thickness, μm	210.0 ± 81.6	213.9 ± 103.6	235.4 ± 118.7	0.422 ^‡^
Central fovea thickness, μm	203.0 ± 65.7	297.5 ± 179.1	272.7 ± 121.7	0.534 ^‡^
CNV area, mm^2^	2.2 ± 1.3	1.9 ± 2.5	-	0.816 ^∥^
SRF existence, no. of eyes	7	13	4	0.561 ^†^
SRF height in eyes with SRF, μm	135.7 ± 76.9	100.8 ± 62.5	106.0 ± 56.4	0.492 ^‡^

BCVA: best-corrected visual acuity; IOP: intraocular pressure; PED: pigment epithelial detachment (serous or vascularized); CNV: choroidal neovascularization; SRF: subretinal fluid. * one-way ANOVA; ^†^ Chi square test; ^‡^ Kruskal–Wallis test; ^§^ Fisher’s exact test; ^∥^ independent *t*-test.

**Table 3:** Parameters of OCT in eyes with neovascular age-related macular degeneration according to subtype of choroidal neovascularization (CNV).

	Type 1 CNV	Type 2 CNV	Type 3 CNV	*p*-Value
No. of eyes	34	9	9	-
Sex (female, %)	13 (38.2)	2 (22.2)	9 (100)	0.01 *
Mean age, years	73.6 ± 9.2	71.56 ± 8.1	73.4 ± 8.6	0.622 ^†^
No. of treatment naive eyes	2	3	2	0.052 *
No. of intravitreal injection	6.3 ± 4.5	5.3 ± 5.7	2.7 ± 2.1	0.121 ^†^
IOP, mmHg	14.09 ± 2.84	15.25 ± 3.96	14.77 ± 2.24	0.232 ^†^
BCVA, logMAR	0.52 ± 0.43	0.53 ± 0.29	0.53 ± 0.24	0.643 ^‡^
Disease duration, months	19.4 ± 14.1	13.8 ± 16.3	10.7 ± 14.1	0.064 ^‡^
PED max height, μm	168.7 ± 151.9	147.4 ± 78.2	180.1 ± 171.5	0.822 ^‡^
PED max width, μm	2466.7 ± 1,398.9	2543.8 ± 1,001.3	1733 ± 1,470.8	0.310 ^‡^
Choroidal thickness, μm	232.5 ± 101.1	234.2 ± 90.5	139.6 ± 51.1	0.016 ^§^
Center fovea thickness, μm	252.6 ± 119.3	198.5 ± 75.7	349.2 ± 214.8	0.422 ^‡^
CNV area, mm^2^	1.95 ± 1.28	3.52 ± 3.41	0.51 ± 0.83	0.006 ^∥^
SRF height in eyes with SRF, μm	100.0 ± 51.9	104.1 ± 48.9	165.0 ± 96.2	-
SRF existence, no. of eyes	14	6	4	0.466 *

BCVA: best-corrected visual acuity; IOP: intraocular pressure. PED: pigment epithelial detachment (serous or vascularized); CNV: choroidal neovascularization; SRF: subretinal fluid. * Fisher’s Exact test; ^†^ One-way ANOVA; ^‡^ Kruskal-Wallis with post-hoc Analysis, ^§^ Type 1–Type 3, *p* = 0.019; ^∥^ Type 2–Type 3, *p* = 0.005.

**Table 4:** Choroidal neovascularization (CNV) visualization group, CNV detection sensitivity, and CNV detection score according to CNV subtype.

	Total (*n* = 52)	Type 1 CNV (*n* = 34)	Type 2 CNV (*n* = 9)	Type 3 CNV (*n* = 9)	*p*-Value
Group A (well-circumscribed)	18	10	5	3	
Group B (moderately circumscribed)	24	15	4	5	
Group C (poorly circumscribed)	10	9	0	1	
Detection sensitivity, %	80.7 (42/52)	73.5 (25/34)	100 (9/9)	88.9 (8/9)	0.159 *
CNV detection score, point	2.15 ± 0.72	2.03 ± 0.76	2.56 ± 0.53	2.22 ± 0.63	0.158 ^†^

* Fisher’s exact test, ^†^ Kruskal-Wallis.

**Table 5:** Parameters of OCT according to choroidal neovascularization (CNV) group in eyes with type 1 CNV.

	Group A	Group B	Group C	*p*-Value
No. of eyes	10	15	9	
Mean age	77.3 ± 7.60	71.6 ± 10.2	72.9 ± 9.3	0.329 ^†^
Sex (female, %)	6 (60)	3 (30)	4 (44.5)	0.158 ^†^
BCVA, logMAR	0.66 ± 0.51	0.40 ± 0.29	0.53 ± 0.52	0.364 ^†^
IOP, mmHg	13.2 ± 2.8	15.1 ± 2.7	15.8 ± 4.3	0.195 *
No. of treatment naive eyes	0	2	0	0.492 ^†^
No. of intravitreal injection	5.0 ± 2.7	7.1 ± 5.1	6.3 ± 4.9	0.541 ^‡^
Disease duration, months	15.6 ± 10.4	21.6 ± 15.6	19.8 ± 15.2	0.597 *
PED max height, μm	1193.3 ± 214.6	144.9 ± 122.9	181.1 ± 120.6	0.72 ^‡^
PED max width, μm	2729.2 ± 1473.2	2577.2 ± 1386.1	1990.9 ± 1380.6	0.489 ^‡^
Choroidal thickness, μm	205.1 ± 73.9	239.5 ± 111.0	251.2 ± 114.2	0.588 ^‡^
Center fovea thickness, μm	214.6 ± 67.3	264.8 ± 140.1	274.7 ± 128.9	0.491 ^‡^
CNV area, mm^2^	2.31 ± 1.27	1.70 ± 1.27	-	0.249
SRF existence, number of eyes	3	7	4	0.747 ^†^
SRF height in eyes with SRF, μm	139.3 ± 57.7	79.7 ± 43.0	106.0 ± 56.5	0.532 ^‡^

BCVA: best-corrected visual acuity; IOP: intraocular pressure; PED: pigment epithelial detachment (serous or vascularized); CNV: choroidal neovascularization; SRF: subretinal fluid. * One-way ANOVA, ^†^ Fisher’s exact test; ^‡^ Kruskal-Wallis test.
